# Mosquito, Bird and Human Surveillance of West Nile and Usutu Viruses in Emilia-Romagna Region (Italy) in 2010

**DOI:** 10.1371/journal.pone.0038058

**Published:** 2012-05-30

**Authors:** Mattia Calzolari, Paolo Gaibani, Romeo Bellini, Francesco Defilippo, Anna Pierro, Alessandro Albieri, Giulia Maioli, Andrea Luppi, Giada Rossini, Agnese Balzani, Marco Tamba, Giorgio Galletti, Antonio Gelati, Marco Carrieri, Giovanni Poglayen, Francesca Cavrini, Silvano Natalini, Michele Dottori, Vittorio Sambri, Paola Angelini, Paolo Bonilauri

**Affiliations:** 1 Istituto Zooprofilattico Sperimentale della Lombardia e dell’Emilia Romagna “B. Ubertini” (IZSLER), Brescia, Italy; 2 Regional Reference Centre for Microbiological Emergencies (CRREM), S. Orsola-Malpighi University Hospital, Bologna, Italy; 3 Centro Agricoltura Ambiente “G. Nicoli” (CAA), Crevalcore, Italy; 4 Servizio Veterinario Azienda USL Modena, Mirandola, Italy; 5 Alma Mater Studiorum – Università di Bologna, Bologna, Italy; 6 DG Sanità e Politiche Sociali, Regione Emilia-Romagna, Bologna, Italy; Blood Systems Research Institute, United States of America

## Abstract

**Background:**

In 2008, after the first West Nile virus (WNV) detection in the Emilia-Romagna region, a surveillance system, including mosquito- and bird-based surveillance, was established to evaluate the virus presence. Surveillance was improved in following years by extending the monitoring to larger areas and increasing the numbers of mosquitoes and birds tested.

**Methodology/Principal Findings:**

A network of mosquito traps, evenly distributed and regularly activated, was set up within the surveyed area. A total of 438,558 mosquitoes, grouped in 3,111 pools and 1,276 birds (1,130 actively sampled and 146 from passive surveillance), were tested by biomolecular analysis. The survey detected WNV in 3 *Culex pipiens* pools while Usutu virus (USUV) was found in 89 *Cx. pipiens* pools and in 2 *Aedes albopictus* pools. Two birds were WNV-positive and 12 were USUV-positive. Furthermore, 30 human cases of acute meningoencephalitis, possibly caused by WNV or USUV, were evaluated for both viruses and 1,053 blood bags were tested for WNV, without any positive result.

**Conclusions/Significance:**

Despite not finding symptomatic human WNV infections during 2010, the persistence of the virus, probably due to overwintering, was confirmed through viral circulation in mosquitoes and birds, as well as for USUV. In 2010, circulation of the two viruses was lower and more delayed than in 2009, but this decrease was not explained by the relative abundance of *Cx. pipiens* mosquito, which was greater in 2010. The USUV detection in mosquito species confirms the role of *Cx. pipiens* as the main vector and the possible involvement of *Ae. albopictus* in the virus cycle. The effects of meteorological conditions on the presence of USUV-positive mosquito pools were considered finding an association with drought conditions and a wide temperature range. The output produced by the surveillance system demonstrated its usefulness and reliability in terms of planning public health policies.

## Introduction

In recent years, there have been increasing global reports of diseases due to arboviruses (arthropod-borne viruses) [1], [2], [3].

The detection of viruses in invertebrate vectors and vertebrate reservoir hosts is an important part of public health surveillance systems that allows analysis of the intensity and seasonality of viral circulation in the environment; it provides date for the timely planning of preventive measures [4] such as blood donation screening.

Surveillance systems should be established to monitor specific arthropod and vertebrate species, according to the target virus and environmental conditions. Following this indications, a surveillance system targeting West Nile virus (WNV) (Flaviviridae, Flavivirus) should mainly include *Culex* mosquitoes and wild birds, such as corvids [5], major vectors and reservoirs of the virus, respectively. A surveillance system with these characteristics was set up in the Emilia-Romagna region during 2008, after detection of WNV in the region [6], [7], [8]. Surveillance was increased in subsequent years, extending the monitored area and increasing the number of sampled birds and mosquitoes [9], [10].

In 2009 this system detected Usutu virus [11], another flavivirus of the Japanese encephalitis antigenic complex [12], phylogenetically closely related to WNV [13], [14].

While WNV activity was reported in Europe since the 1960s [1], [15], [16] and several studies implied the silent circulation of this virus in Europe [17], [18], USUV was first confirmed in Europe in Vienna in 2001 [19] and subsequently spread to neighboring countries, Hungary [20], Italy [21], Switzerland [22] Czech Republic [23], and Poland [24]; USUV was also detected in other European countries, such as Spain [25] and England [17].

WNV and USUV show several common characteristics: the main vectors of both viruses are largely ornithophilic mosquitoes, mainly of the genus *Culex*; wild birds are principal reservoirs of WNV and potentially also of USUV; both viruses can be pathogenic for these host species [1], [3], [5], [26], [27], [28], [29], [30].

While the risks of WNV for human health are well recognized, the medical importance of USUV is not fully understood. This virus appears to be pathogenic to humans, as demonstrated by some case reports: USUV-related illness in two immunocompromised patients reported in the Emilia-Romagna region in summer 2009 [31], [32]; detection of USUV in cerebrospinal fluid from three patients with meningoencephalitis [33]; isolation of USUV from two patients in Africa, one with fever and rash, and one 10-year-old with fever and jaundice [34]; and detection in a patient with rash in Austria [35]. Furthermore USUV IgG-specific antibodies were recently identified in four healthy blood donors with no history of flavivirus infection in Italy [36].

Due to the lack of ecological data, the USUV cycle is often assimilated to the WNV cycle, but some ecological differences emerged between the two viruses during their simultaneous detections in Emilia-Romagna region in 2009. While WNV was only detected in *Culex pipiens*, USUV was also found in *Aedes albopictus* mosquito pools; moreover, unlike USUV, WNV was more abundant in rural areas [10].

The main aim of the surveillance system was the early detection of viral circulation. In addition to this the obtained data allowed to evaluate the differences and similarities between the two virus cycles and the role of environmental and climatic factors in the diffusion of the viruses.

## Results

### Viruses in Mosquitoes

During the period May 31-October 11 2010, a total of 438,558 mosquitoes, grouped in 3,111 pools, were collected and analyzed. The most abundant species was *Cx. pipiens* (90.9% of total mosquitoes), followed by *Ae. caspius* (4.1%), *Ae. vexans* (4.0%), *Ae. albopictus* (0.4%) and *Cx. modestus* (0.3%) (Table 1). Other sampled species, but at very low density (under 0.2%), were *Ae. detritus, Ae. geniculatus, Anopheles maculipennis* s.l., *An. plumbeus, Coquillettidia richiardii* and *Culiseta annulata* (Table 1).

**Table 1:** Total number of specimens, PCR pools and PCR-positive pools collected for every mosquito species in 2010 survey.

SPECIES	N	%	Pools	WNV/+	USUV/+
*Aedes albopictus*	1,855	0.4	131		2
*Aedes caspius* *	18,135	4.1	367		
*Aedes detritus* *	46	<0.2	2		
*Aedes geniculatus* *	532	0.1	5		
*Aedes vexans*	17,697	4.0	185		
*Anopheles maculipennis* s.l.	167	<0.2	17		
*Anopheles plumbeus*	15	<0.2	4		
*Coquillettidia richiardii*	167	<0.2	4		
*Culiseta annulata*	1	<0.2	1		
*Culiseta spp.*	1	<0.2	1		
*Culex modestus*	1,107	0.3	27		
*Culex pipiens*	398,835	90.9	2,367	3	89
Total	438,558		3,111	3	91

* *Ochlerotatus* taxon was considered an *Aedes* subgenus [60].

**Figure 1:**
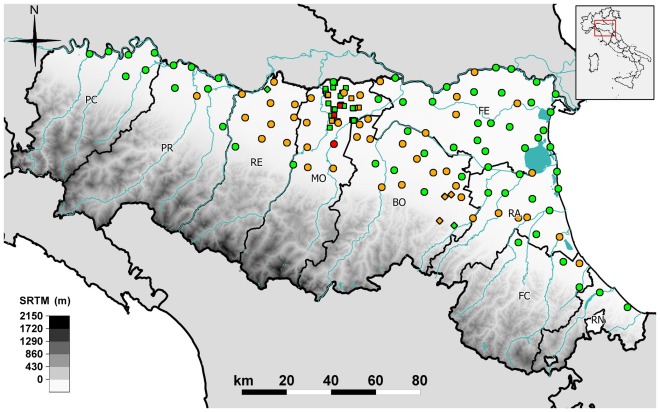
Emilia-Romagna regional map of mosquito sampling stations and locations of PCR–positive pools. Circle, plan trap; square, Modena trap; diamond, extra-plan trap; red, WNV– and USUV–positive station; orange, USUV–positive station; green, negative station. Province abbreviations: PC, Piacenza; PR, Parma; RE, Reggio Emilia; MO, Modena; BO, Bologna; FE, Ferrara; RA, Ravenna; FC, Forlì Cesena; RN, Rimini.

A total of 393,058 specimens were sampled by plan traps, while 41,327 mosquitoes were sampled by Modena traps and 4,173 by extra-plan traps. The distribution of these different traps is shown in Figure 1. A comparison of mosquito abundance between 2009 and 2010, considering the same activation period, was possible for 14 plan traps (8 located in Bologna province and 6 in Ferrara province) and 6 Modena traps; the average number of mosquitoes per night was higher in 2010 than in 2009 (average mosquitoes/night, 329.62, and 180.5 respectively, p<0.05).

During the survey, 3 pools of *Cx. pipiens* were found to be WNV-positive (positive results confirmed by the National Centre for the Study of Exotic Diseases, CESME, Teramo, Italy); 89 pools of *Cx. pipiens* and 2 of *Ae. albopictus* were USUV-positive (Table 1). Furthermore, sequence data showed the presence of mosquito-only flavivirus RNA in one pool of *Ae. albopictus* sampled in Ferrara province on August 27.

All of the 3 WNV-positive pools were sampled in Modena province, one by a plan trap on August 26 (bi-weekly maximum likelihood estimation, MLE = 0.23, confidence interval, CI = 0.01–1.1) and two in Modena traps during the same bi-weekly period on August 23 and 30 (bi-weekly MLE = 0.15, CI = 0.03–0.49). The last two pools were also USUV-positive, and WNV-MLE values were lower than the 2009 MLE minimum values obtained in the same area [5], [10].

**Figure 2:**
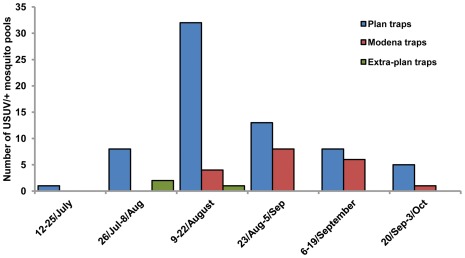
Seasonal distribution of positive USUV mosquito pools (bi-weekly collection) per kind of trap.

Of the total 91 USUV-positive mosquito pools, 67 were sampled in plan-traps (Figure 2). The earliest USUV-positive pool was sampled in Modena province on July 22, about one month before the collection of the first WNV-positive pool (Figure 2). The last four pools that tested positive for USUV were sampled at three different sites (from Bologna and Modena provinces) on September 23 (Figure 2). The highest bi-weekly MLE of USUV infection rates of *Cx. pipiens* estimated from pooled samples per province was detected in Forlì-Cesena province between August 23 and 25 (MLE = 3.1, CI = 0.2–17); the longest period of circulation occurred in Modena province, from July to September, with MLE values ranging from 0.1 to 1.5 (Table 2). No USUV-positive pools were sampled in Piacenza and Rimini provinces. The two *Ae. albopictus* USUV-positive pools were sampled on August 25 in Bologna province by an extra-plan trap (insufficient data to calculate MLE) and on September 9 in Modena province by a Modena trap (MLE = 12.59, CI = 0.69–68.83).

**Table 2:** Bi-weekly maximum likelihood estimates (95% CI) of USUV infection rates in *Culex pipiens* estimated at province level.

	BO	FC	FE	MO	PC	PR	RA	RE	RN
31/May-13/Jun	0 (0–0.6)	0 (0–2)	0 (0–0.3)	0 (0–0.5)		0 (0–0.7)	0 (0–0.7)	0 (0–1)	0 (0–15.2)
14–27/Jun	0 (0–0.4)	0 (0–6.1)	0 (0–0.2)	0 (0–0.4)	0 (0–0.7)	0 (0–0.6)	0 (0–0.7)	0 (0–0.7)	0 (0–39.5)
28/Jun-11/Jul	0 (0–0.4)	0 (0–1.8)	0 (0–0.2)	0 (0–0.4)	0 (0–0.8)	0 (0–0.7)	0 (0–0.9)	0 (0–0.5)	0 (0–8)
12–25/Jul	0 (0–0.6)	0 (0–2.5)	0 (0–0.2)	0.1 (0–0.5)	0 (0–0.8)	0 (0–0.6)	0 (0–1.1)	0 (0–0.5)	0 (0–16.8)
26/Jul-8/Aug	0.1 (0–0.6)	0 (0–3.9)	0.1 (0–0.4)	0.4 (0.1–1.1)	0 (0–0.8)	0 (0–0.7)	0 (0–1.1)	0.4 (0.1–1.2)	0 (0–5.7)
9–22/Aug	1.5 (0.7–2.9)	0 (0–6.7)	0.1 (0–0.6)	1.1 (0.5–2.2)	0 (0–1.1)	0.3 (0–1.6)	1.1 (0.4–2.6)	1.5 (0.8–2.8)	0 (0–231.2)
23/Aug-5/Sep	1 (0.4–2.2)	3.1 (0.2–17)	0.2 (0–0.7)	1.2 (0.5–2.7)	0 (0–1.1)	0 (0–2)	0 (0–2.3)	0 (0–0.8)	0 (0–14.9)
6–19/Sep	0.3 (0.1–1.1)	0 (0–8.4)	0.4 (0.1–1)	0.3 (0–1.7)	0 (0–2.6)	0 (0–1.9)	0.4 (0–2.1)	0.5 (0–2.5)	0 (0–7.9))
20/Sep-3/Oct	0.5 (0–2.5)	0 (0–65.9)	1.5 (0.4–4.1)	1.5 (0.1–7.7)		0 (0–5.7)	0 (0–3.6)	0 (0–3.3)	

Province abbreviations: BO, Bologna; FC, Forlì-Cesena; FE, Ferrara; MO, Modena; PC, Piacenza; PR, Parma; RA, Ravenna; RE, Reggio Emilia; RN, Rimini.

### Viruses and Pathology in Wild Birds

A total of 1,130 wild birds, collected within the specific wildlife population control programs between May and October, were tested by the three described polymerase chain reactions (PCRs). These birds belonged to 7 species, with 3 corvid species being most common: European Magpies (*Pica pica*; 53.1%), Hooded Crows (*Corvus cornix*; 28%) and Eurasian Jays (*Garrulus glandarius*; 7.5%) (Table 3).

**Table 3:** Birds tested in active and passive surveillance (A/P) with WNV- and USUV-positive specimens.

Order	Species	Common name	N (A/P)	WNV/+ (A/P)	USUV/+ (A/P)
Passeriformes					
	*Pica pica*	European Magpie	601 (600/1)	1 (1/0)	1 (1/0)
	*Corvus cornix*	Hooded Crow	317 (316/1)		
	*Corvus frugilegus*	Rook	12 (12/0)		
	*Garrulus glandarius*	Eurasian Jay	88 (85/3)	1 (1/0)	1 (0/1)
	*Turdus merula*	Eurasian Blackbird	11 (0/11)		5 (0/5)
	*Sturnus vulgaris*	European Starling	56 (51/5)		
Pelecaniformes					
	*Phalacrocorax carbo*	Great Cormorant	23 (21/2)		
Columbiformes					
	*Columba livia domestica*	Domestic Pigeon	55 (45/10)		
	*Streptopelia decaocto*	Collared Dove	11 (0/11)		1 (0/1)
Strigiformes					
	*Asio otus*	Long-eared Owls	4 (0/4)		2 (0/2)
Caprimulgiformes					
	*Caprimulgus europaeus*	Nightjar	1 (0/1)		1 (0/1)
Galliformes					
	*Alectoris rufa*	Partridge	1 (0/1)		1 (0/1)
Other bird species*			96 (0/96)		
Total			(1130/146)	2 (2/0)	12 (1/11)

* Other bird species tested in passive surveillance (number of specimens): Apus apus (16), Larus michahellis (14), Athene noctua (6), Falco tinnunculus (5), Otus scops (5), Picus viridis (5), Hirundo rustica (4), Passer domesticus (4), Ardea cinerea (2), Buteo buteo (2), Carduelis carduelis (2), Ciconia ciconia (2), Larus ridibundus (2), Perdix perdix (2), Scolopax rusticola (2), Accipiter nisus (1), Anas platyrhynchos (1), Ardea purpurea (1), Ardea spp. (1), Asio spp. (1), Carduelis chloris (1), Carduelis spinus (1), Cuculus canorus (1), Dendrocopos spp. (1), Egretta garzetta (1), Erithacus rubecula (1), Falco subbuteo (1), Fringilla coelebs (1), Fulica atra (1), Merops apiaster (1), Passer montanus (1), Phasianus colchicus (1), Phoenicurus phoenicurus (1), Podiceps cristatus (1), Recurvirostra avosetta (1), Sterna hirundo (1), Strix aluco (1), Upupa epops (1).

Two birds tested positive for WNV: one European Magpie collected on August 1 in Bologna province and one Eurasian Jay collected on August 4 in Modena province (positive results confirmed by CESME). Moreover, one European Magpie, collected in Bologna province on July 28, was USUV-positive (Table 3).

Another 146 birds, belonging to 49 species, were collected from wildlife rehabilitation centers (WRCs) or were found dead on the field. No WNV-positive samples were detected among these birds but 11 were USUV-positive, including five Eurasian Blackbirds (*Turdus merula*), two of which were captive birds, two came from WRCs and one was found dead on the field. The remaining 6 birds were one Eurasian Jay, one Collared Dove (*Streptopelia decaocto*), one Nightjar (*Caprimulgus europaeus*), one Partridge (*Alectoris rufa*) all from WRCs and two Long-eared Owls (*Asio otus*), one from WRCs and one found death on the field (Table 3). All that birds were necropsied. In two Eurasian Blackbirds (*Turdus merula*) and in one Long-eared Owl (*Asio otus*) resulted infected by USUV a marked splenomegaly was observed. No gross lesions were recorded from other USUV-positive birds.

### Viruses in Humans

Between July 31 and December 1, 30 patients with clinical indications of meningoencephalitis were evaluated by RT-PCR for the presence of WNV or USUV genome in plasma/serum and/or cerebrospinal fluid (CSF). All of the subjects lived in the Emilia-Romagna region. The male/female ratio was 16∶14 and the median age was 48 (range 17 to 83 years). No patients tested positive for either WNV or USUV in plasma/serum or CSF. In addition, a total of 1,053 blood bags, from presumptive WNV circulation area, were tested by nucleic acid amplification technique (NAAT) assay.

### Virus Sequences

The WNV-positive PCR amplicons, obtained from the two birds (GenBank (GB) accession numbers: JF714987-88) and the three mosquito pools (GB accession numbers: JF714984-86) presented identical sequences. These sequences showed a high degree of identity, ranging from 99.8% to 100% (with a maximum of 3 differences on 407 bp considered), with other sequences obtained from mosquitoes [5], [6], human (GB accession number: GU011992) and birds (GB accession numbers: FJ472947, FJ483548-49, JF719065, JF7119067-68) in the 2008–2009 Italian outbreak.

**Figure 3:**
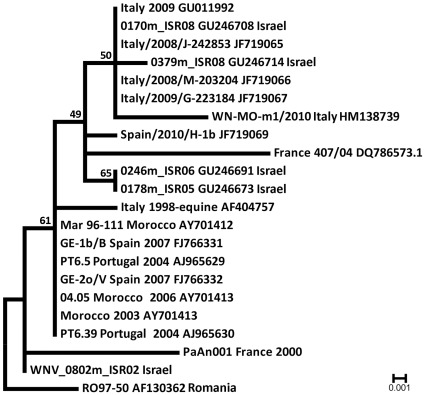
Molecular Phylogenetic analysis by Maximum Likelihood method based on the Kimura two-parameter model of the WNV sequences of amplified fragments and homologous fragment obtained in GenBank library. The tree with the highest log likelihood (−720.3421) is shown. Initial tree for the heuristic search were obtained automatically by Neighbor-joining method with Maximum Composite Likelihood distance matrix. The analysis involved 22 nucleotide sequences, there were a total of 409 positions in the final dataset. Evolutionary analyses were conducted in MEGA5 [65], the tree was rooted in RO97 50. Percentages of bootstrap test (1000 replicates) are shown next to the branches.

Moreover, these sequences showed a higher degree of identity, of 99.0% (4 differences on 409 bp considered), for homologous sequences obtained from mosquito pools sampled in Israel in 2008 (GB accession numbers: GU246708, GU246710) than for other European sequences present in the GB database, including the sequence obtained from a horse in the 1998 Italian outbreak (GB accession number: AF404750) (Figure 3).

A total of 139 sequences were obtained from USUV-positive pools, 79 of which were from *Cx. pipiens* mosquito, 1 from *Ae. albopictus* mosquito and 15 from birds.

The 51 sequences obtained for flavivirus-genus PCR from 48 *Cx. pipiens* mosquito pools (GB accession numbers: JF834546-61, JF834563-94) and 3 birds (GB accession numbers: JF834562, JF834595, JF834596) showed an identity range from 98.5 to 100% on 199 positions which were compared. In 6 sequences, a mutation can lead to different amino acid changes in translated protein sequence (Table 4). These sequences were also very similar to sequences obtained in Emilia-Romagna in 2009 [10].

**Table 4:** Number of USUV sequences obtained in this study with mutation producing an amino acidic modification in the translated protein sequence.

	N of seq. with mutation/total	Mutation	Position respect EF206350	AA change	Position respect EF206350
*E*	1/87	G>A	1353	M>I	451
	1/87	C>T	1466	A>V	489
	24/87	A>G	1477	T>A	493
	2/87	A>G	1531	I>V	511
	6/87	G>A	1558	G>R	520
*NS5*	1/51	G>C	9086	G>A	3029
	1/51	G>A	9250	E>K	3084
	3/51	C>T	9259	H>Y	3087
	1/51	G>T	9304	D>Y	3097

The 87 sequences obtained by specific USUV PCR, 12 from birds (GB accession numbers: JF834597, JF834617-18, JF834657-62, JF834669, JF834683), 75 from *Cx. pipiens* pools (GB accession numbers: JF834598-16, JF834619-52, JF834654-56, JF834663, -68, JF834670-82) and one from *Ae. albopictus* pool (GB accession number: JF834653) showed an identity ranging from 98.3 to 100%, on 354 positions which were compared. Five mutations in the obtained sequences can lead to changes in sequence of translated proteins (Table 4); in particular, 24 sequences showed a transition (from A to G) in position 1477 of the retro transcribed USUV genome (GB accession number: EF206350), producing a substitution of threonine with alanine in position 493 of the derived translated protein (Table 4).

The consensus sequences of both PCR products showed the highest scores in BLAST analysis with the 2009 Italian sequences [10], and also a high identity with the two USUV strains isolated in Budapest (GB accession number: EF206350) and in Vienna (GB accession number: AY453411), with a 96.5% and 99% identity for partial *NS5* gene and partial *E* gene, respectively.

None of the 14 homologous sequences present in GB database (from Austria, Hungary and South Africa) showed the amino acidic change led by mutation of sequences obtained in the present study, and all the Italian sequences showed tyrosine in position 392 rather than an aspartic acid, which was present in all the other sequences.

### Influence of Ecological Factors in WNV and USUV Cycle

According to land use classifications, 71 of the regional plan traps were positioned in rural areas, 16 in urban areas, 10 in wetlands, 4 in woodland and 1 in a green area (Table 5).

**Table 5:** Details of USUV-positive pools of *Cx. pipiens* mosquito according to land use categories.

	N of traps (%)	N of *Cx. pipiens*pools (%)	Mean N *Cx.* *pipiens*/trap	N of USUV/+pools (%)	% of +/pool per area
Rural areas	71 (69.6)	1530 (75.4)	729.9	54 (80.6)	3.5
Urban areas	16 (15.7)	201 (9.9)	294.0	8 (11.9)	4.0
Wetlands	10 (9.8)	231 (11.4)	811.3	3 (4.5)	1.3
Woodlands	4 (3.9)	60 (3)	443.1	2 (3)	3.3
Green areas	1 (1)	8 (0.4)	46.6		
TOTAL	102	2030		67	

The largest proportion of the sampled *Cx. pipiens* pools (1,530∶75.4%) came from the rural areas; 231 (11.4%) pools were sampled in the wetlands, 201 (9.9%) in the urban areas, 60 (3%) in the woodlands and 8 (0.4%) in the green areas (Table 5).

Among the 3 WNV-positive pools, 2 were sampled in rural areas and one in an urban area. Due to the few positive stations obtained, no further analysis was possible.

Regarding the USUV-positive pools these were evenly sampled in different environments, and their abundance reflected the abundance of *Cx. pipiens* pools sampled); for example 80.6% of these positive pools were sampled in rural areas, where 75.4% of *Cx. pipiens* pools were captured, similarly the 11.9% of positive pools came from urban areas, where 9.9% of pools were sampled (Table 5). Interestingly the probability of obtaining a positive pool is not related with the abundance of *Culex* mosquitoes and is highest in urban areas, but these data were statistically not significant (p>0.10).

Among the screened parameters, from June to September, the total rainfall and total evapotranspiration, were significantly different at sites in which at least one positive mosquito pool was obtained compared to negative stations (p<0.05). These two parameters were resumed by hydro-climatic balance (HCB) that was significantly lower at positive sites (Table 6). Also, temperature range, represented by the difference between the maximum and minimum temperatures, was significantly wider at positive stations compared to the negative stations, but average temperature did not differ significantly (Table 6). The minimum daily humidity was significantly lower in positive stations; maximum and average relative humidity did not show significant differences. The average number of *Cx. pipiens* mosquitoes tested and NDVI were higher at USUV-positive stations than in negative ones, but these differences were not significant (Table 6).

**Table 6:** Evaluation of variations in climatic conditions (average from June to September) and *Cx. pipiens* numbers at stations with/without at least one USUV-positive pool.

	Positive stationaverage (SD)	Negative stationaverage (SD)	ANOVA testp-value
Total rainfall (mm)	241.3 (33.7)	265.9 (52.3)	<0.01*
Total evapotranspiration (mm)	566.0 (24.9)	553.4 (26.1)	<0.05*
Hydro-climatic balance (mm)	−324.6 (44.5)	−287.5 (58.0)	<0.01*
Average temperature (°C)	22.2 (0.3)	22.2 (0.3)	>0.1
Minimum temperature (°C)	16.3 (0.7)	16.6 (0.7)	<0.05*
Maximum temperature (°C)	27.9 (0.5)	27.7 (0.5)	<0.05*
Temperature range (°C)	11.6 (1.0)	11.1 (1.0)	<0.05*
Relative humidity average (%)	66.7 (2.9)	67.2 (2.8)	>0.1
Minimum relative humidity (%)	44.4 (2.1)	45.5 (2.3)	<0.05*
Maximum relative humidity (%)	87.9 (3.7)	87.6 (3.8)	>0.1
Normalized Difference Vegetation Index (NDVI)	0.58 (0.1)	0.55 (0.1)	>0.1
Average of examined *Cx. pipiens* spec.	443.9 (217.7)	383.9 (207.6)	>0.1

### Geospatial and Point Pattern Analysis

**Figure 4:**
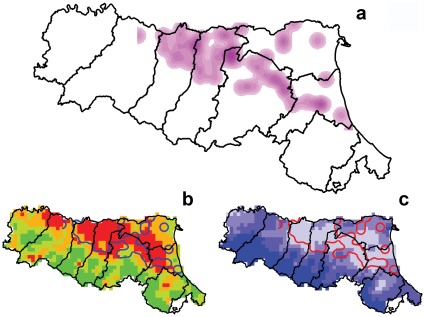
USUV “hot spots” obtained by kernel density estimation of positive mosquito pools (a); overlaying of 95% contour perimeter and quartile maps of temperature range (b); and of hydro-climatic balance (c); from July to September 2010.

Nearest Neighbor Analysis (NNA) of USUV-positive traps confirmed that the spatial pattern was clustered (R = 0.68, Z-Score =  −5.67), allowing the analysis to proceed with kernel density estimation (KDE). The dissemination surfaces of USUV represented by a KDE map, derived from the 102 fixed traps (Figure 4), identified primarily virus circulation “hot spots” in the north of the provinces of Modena and Reggio-Emilia and secondarily (less intense) hot spots between the Bologna and Ravenna provinces and in the north of Ferrara province. It was found that 57% of the circulation area (Figure S1) was within the upper quartile of the temperature range (Figure 4), and that 56% and 41% of that area occurred within the lower quartile of the hydroclimatic balance and in the lower quartile of the minimum daily humidity, respectively (Figure 4, Figure S1).

## Discussion

The 2010 survey data confirm the persistent circulation of WNV and USUV in the Po Plain, after their detection in previous years [6], [7], [10].

The high homology between sequences detected in birds and mosquitoes suggest that, for both viruses, a single strain circulated in 2010 in the surveyed area, as evidenced in the 2009 season [10]. Moreover, these data highlight a high rate of identity between the 2010 sequences and those obtained in the previous years, supporting a probable USUV and WNV overwintering between 2009 and 2010, as hypothesized for WNV in 2008–2009 [10], [37].

The obtained WNV sequences show a high rate of identity with 2008 Israeli sequences, suggesting a possible common origin of WNV strains circulating in the two countries in recent years (Figure 4). Interestingly, the 2008–2010 sequences from Emilia-Romagna have a lower rate of identity for the 1998 Italian sequence than for sequences from other European countries, like those detected in Spain in 2010 and in France in 2004 (GB accession numbers: JF719069, DQ786573), suggesting that the introduction of a new virus strain, probably from different areas of the Mediterranean basin [38], occurred at least twice in Italy since 1998.

Unfortunately, there are few GB sequences for USUV; among these, both the *E* and *NS5* USUV sequences show the highest nucleotide identity rates with strains originating from Vienna and Budapest, compared to South African sequences, implying that the USUV strain detected in central Europe is the same as that circulating in northern Italy. Notably, in the homologous tract of the *NS5* gene, a common mutation is present between South African and three Austrian translated amino acid sequences (valine instead of a methionine in position 451 of South African strains and in GB sequences EF393680, EF393679, EF078301), indicating the affinity between these strains, while the amino acid mutations present only in the Italian sequences (Table 4) may have been acquired by the virus during its southern expansion.

The sequences obtained from flavivirus-positive PCR, not related to WNV or USUV, are due to the presence of a mosquito-only flavivirus, isolated from *Ae. albopictus* in Japan, and already detected during previous entomological surveys performed in Italy [9], [10], [39].

The finding that 90.9% of the 438,558 tested mosquitoes belong to *Cx. pipiens,* the only species found WNV-positive in surveys to date, confirms this species as the main WNV vector in northern Italy. The survey data did not indicate involvement of other mosquito species in the WNV cycle. The USUV data also indicate *Cx. pipiens* as the principal vector of this virus, accounting for 89 of 91 positive pools. The finding of 2 USUV-positive pools referring to *Ae. albopictus* supported the 2009 evidence [10], indicating this species as a possible USUV vector. Further experimental studies are needed to confirm the vector competence and the role of the Asian Tiger mosquito (*Ae. albopictus*) in the USUV cycle.

The circulation of both viruses was lower in 2010 than in 2009, as shown by the MLE values when compared by areas, with the 2010 bi-weekly maximum values, between August and September, of 1.5 and 0.4 respectively in Bologna and Ferrara provinces (Table 2), and maximum values of 2.9 and 0.7, respectively, during the same period of 2009 [10].

As well the data obtained from mosquitoes sampled in plan traps indicated a delayed virus circulation in the 2010 compared to the 2009 seasons, as evidenced by the collection dates of the first positive pools for both WNV (August 23 in 2010 and July 21 in 2009) and USUV (July 27 in 2010 and July 8 in 2009).

The decrease in viral activity observed in 2010 in comparison with 2009 is not explained by the density of *Cx. pipiens* which, on the contrary, was higher in 2010 than in 2009, as also evidenced by data collected from 21 traps employed in Bologna province to monitor mosquito abundance in the two survey years (from May to September), with an average of 386.6 *Cx. pipiens*/night in 2010 and of 259.0 in 2009 [40]. Moreover, in comparing the mean collection of *Cx. pipiens*/trap per night in USUV-positive *vs.* USUV-negative stations significant differences were not observed. These observations support the absence of a direct relationship between mosquito abundance and USUV activity.

The relative number of USUV-positive mosquito pools, sampled at regular intervals from an evenly distributed network of traps, (the plan traps, Figure 1), allowed the evaluation of the possible influence of climatic conditions. This analysis indicated that the presence of USUV in mosquitoes was favored by drought conditions, evidenced by the lower hydro-climatic balance (HCB) and lower minimum relative humidity recorded at USUV-positive stations (Table 6). This observation was also supported by geospatial analysis, in which 56% of the 95%-volume KDE contour was within the first quartile area of the HCB map (Figure 4). Similar results were obtained for WNV in previous studies, which evidenced the positive influence of drought conditions on WNV circulation [41], [42], [43], [44]. This parameter was suggested as an influence on ecological dispersal factors, particularly on the concentration of birds and mosquitoes near water sources [43], while another study suggested that drought conditions may affect mosquito bionomics by influencing their vector competence [45]. This study also found a greater temperature range in USUV-positive stations *vs* USUV-negative stations (Table 6, Figure 4). Previous studies reported that daily variation in temperature could affect the vectorial capacity for some mosquito-transmitted pathogens [46], [47]. This effect might be negative or positive depending on the reference mean temperature [47] and a similar effect may also be hypothesized in the case of USUV.

Interestingly, only one bird tested USUV-positive in active surveillance while two birds were WNV-positive. Some USUV serological detections in corvids were reported in Europe [16], [48] but detection by direct tests are scarce [11], [30]. This minor USUV-receptivity among corvids may explain the lack of USUV-positive birds in active surveillance, as corvids were the most examined birds in active surveillance.

No wild birds examined in passive surveillance tested positive for WNV, while 11 tested USUV-positive (Table 3). Three of these birds, two Eurasian Blackbirds and one Long-eared Owl, showed characteristic lesions ascribable to USUV infection [21]. A high USUV pathogenicity was already reported for Eurasian Blackbirds, for which mass mortality due to USUV was described [17], [18], [20], [27], [30] and mortality referred to USUV was also reported in strigiforms [19], [21], [22].

No other *Passeridae* infraorder bird species apart from the Eurasian Blackbirds were found USUV-positive in this study, although several of these birds tested positive in Europe [19], [22], [26], [27], [48]. In relation to other bird species detected positive in this study (Table 3), the Eurasian Jay and the Collared Dove previously tested positive with indirect tests in Austria [48], while no previous references to USUV-positive Partridge or Nightjar were found in the literature.

The presence of 11 positive USUV birds, 3 of which showed sign of probable USUV infection, and the absence of WNV-positive birds in passive surveillance could be the clue of a greater USUV pathogenicity for these animals, compared to WNV, as pointed out by the rarity of bird mortality episodes due to WNV in Europe [5], [15], [27], [28], [49], [50], [51]. The coevolution of birds and WNV in Europe, led by the virus circulation for many years among both migrant and resident birds [50], [52] could have enhanced the possible minor pathogenicity of WNV for wild birds, while a greater USUV pathogenicity for these animals could be due to its recent introduction and the lack of adaptation to the European reservoirs.

In addition, the drastic reduction of WNV circulation between 2009 and 2010, after its 2008 report, is consistent with reports from many European countries, with a progressive reduction in activity following the initial outbreak [50], [51]. So far, this phenomenon seems not to have occurred for USUV, with consistently high activity in the region, despite the first regional serological detection of the virus dating back to 2007 [53].

The ratio of mosquito-positive pools to positive bird differs between WNV (3/2) and USUV (89/11), suggesting the potential involvement of vertebrate reservoirs other than birds in the USUV cycle, but further experimental studies are needed to confirm this hypothesis. Consistently with reduced WNV circulation in 2010, no human cases of meningoencephalitis caused by WNV were observed during this year.

The current data support reports in the literature on the capacity of USUV to become endemic in Europe, highlighting the necessity to survey its presence and diffusion. USUV could have a greater capacity than WNV to spread in urban areas**,** as seems to be evidenced by the high percentage of USUV positive mosquito pools sampled in these environments, and this may be enhanced by the possible vector role of the *Ae. albopictus* mosquito, a marked opportunistic feeder urban mosquito. However, the relevant 2010 USUV circulation did not correspond with the detection of symptomatic USUV infections in humans in the same study period. The recent identification of the first 4 USUV seropositive healthy blood donors in the Emilia-Romagna region [36] is in contrast with the elevated WNV seropositivity found among blood donors in the same area [54] in 2009; this finding suggests that USUV would likely be able to asymptomatically infect humans. The discrepancy in the human epidemiologic data between USUV and WNV and the relevant level of USUV detected in the environment, suggest a lower capability of USUV to infect humans with respect to WNV.

In conclusion, the Emilia-Romagna regional surveillance system is showing good sensitivity, early detection capability and reliability in evidencing WNV and USUV activity: the trap network, covering the whole regional territory, highlighted the areas where viruses activity is higher, while better insight was obtained by placing additional traps in an area of interest, characterized by high viral circulation in the previous year (e.g. Modena traps).

Our results allow assessment of the geographical spread of the viruses and identification of “hot areas” with high virus circulation; mosquito infection rates provide quantifiable information on the intensity of virus transmission and, consequently, information on potential risk to humans and animals [4]. The absence of detected human cases highlights the good sensitivity of the surveillance system, which was able to detect WNV in mosquitoes and birds, while the level of the virus in the environment was likely to be unable to cause human clinical cases and the virus was not detected in checks of 1,053 blood bags. The sensitivity of the integrated surveillance system is also confirmed by the detection of USUV immune response in blood donors in Ferrara province, identified as a virus circulation area.

The obtained data confirm that, with a density of one CO_2_ trap/100 km^2^, detection of arboviruses in mosquitoes usually precedes virus detection in the other surveillance targets and can serve as an early warning tool [4]. It therefore becomes cost-effective to use the data produced by the surveillance system as an indicator of when to start controlling blood bags and to conduct information campaigns on the risks of being bitten by mosquitoes.

Moreover, the introduction of other PCRs to the described surveillance system could allow the detection of other arboviruses, as obtained for Tahyna virus by *Orthobunyavirus*-genus PCR since 2007 in the Emilia-Romagna region [9, unpublished data].

This system can also evaluate the influence of ecological factors on viral circulation, improving both the understanding of environmental spread of viruses and risk assessment related to their presence.

## Materials and Methods

### Survey Area

The survey area, of approximately 10,000 km^2^, was the Emilia-Romagna area of the Pianura Padana, near the Po River; it is the most important Italian floodplain, characterized by intensive agriculture and animal husbandry. The eastern part of the area is located on the Adriatic Sea and is marked by the presence of large natural wetlands (Valli di Comacchio and Po River Delta). The climate is typically sub-continental, gradually becoming sub-Mediterranean towards the coastal part of the region. All of the monitored territories are densely populated, with an abundance of cities, villages, and industrial areas. Industrial activity is highly developed and the Pianura Padana environment is strongly influenced by human activities, defined by intensive agriculture, with few hedges, rare scattered trees and a dense irrigation network; non-cultivated zones are rare (e.g. riverbeds, disused quarries, newly established wetlands). The most common crops are cereals and maize; vineyards or orchards are locally abundant and poplar cultivation is widespread in flood zones near rivers. Pinewood and Mediterranean thicket vegetation are typical of the coastal part of the region.

### Mosquito Collections

Mosquitoes were collected by modified CDC traps baited by CO_2_ (CO_2_ traps) [55]. All traps were georeferenced and worked from approximately 4:00 pm to 10:00 am.

Sampling sites were managed with different modalities: (1) 102 regularly sampled fixed stations, disposed regularly in a 10 km^2^ grid in the surveyed area, activated every 15 days from May 31 to October 1 (plan traps); (2) 36 stations monitored weekly by rotating 10 traps in Modena province, from August 6 to October 11, in an area with intense WNV/USUV circulation in 2009, rotation allowed to sample a station with a single trap about every two weeks (Modena traps); (3) mosquitoes collected for other purpose in 10 single-night sampling stations were also tested (extra-plan traps).

Mosquitoes were identified to species level using morphological characteristics according to four classification keys [56], [57], [58], [59]. The *Ochlerotatus* taxon was considered an *Aedes* sub-genus [60]. Mosquitoes were pooled according to date, location and species, with a maximum of 200 individuals per pool [61]; to avoid cross-contamination due to loose mosquito parts, the pools were obtained by handling specimens individually with tweezers. The pooled mosquitoes were stored in 2 ml (samples of 100 individuals or less) or 4.5 ml (samples of more than 100 individuals) polypropylene cryotubes, and frozen at -80°C. Two 4.3 mm-diameter copper-plated balls (Haendler & Natermann Sport GmbH, Münden, DE) were added to each 2 ml tube, with 4 balls to each 4.5 ml tube. Different amounts of PBS were added to each tube according to the number of stored mosquitoes (0.5 ml up to 30 specimens, 0.9 ml from 31 to 60 specimens, 1.5 ml from 61 to 100 specimens and 3 ml from 101 to 200 specimens). Samples were ground for 40 seconds in a vortex mixer, and then centrifuged at 4000 *g* for 3 minutes; finally, aliquots were collected from the ground samples and submitted to biomolecular analysis. A 200 µl aliquot was collected from the 101–200 specimen homogenates, to optimize time and costs; aliquots from other pools were combined in superpools, with an approach similar to that described in Chisenhall *et al*. [62]: two 100 µl aliquots from 61–100 specimen homogenates, three 67 µl aliquots from 31–60 specimen homogenates, and five 40 µl aliquots from 1–30 specimens homogenate. When a superpool tested positive, each of the original pools comprising in the superpool was tested individually to determine the specific positive sample.

### Bird Collections

Every 1600 km^2^, a monthly sample was collected of approximately 40 wild birds that were caught or shot within specific wildlife population control programs. Monitoring mainly focused on the European Magpie (*Pica pica*), the Hooded Crow (*Corvus cornix*), and the Eurasian Jay (*Garrulus glandarius*), as these corvids are considered pests within the survey area, due to their wide diffusion and eating habits.

Passive surveillance of wild birds was carried out on animals found dead in the field or deceased in wildlife rehabilitation centers. Birds were necropsied, gross lesions have been recorded and for each specimen, organ samples (brain, spleen, heart and kidney) were pooled, ground and submitted to biomolecular analysis; samples from every bird were processed individually. Wild bird active surveillance was carried out following the guidelines contained in the note of the Regional Health Authority PG/2010/93237 of March 31 2010, which regulates the hunting of wild birds for the surveillance of wild-transmitted diseases, so ethics approval was unnecessary.

### Human Collections

The plasma, serum, and CSF samples of 30 subjects with clinical symptoms of meningoencephalitis, collected in Emilia-Romagna between July 31 and December 1 and without diagnosis, were referred to the Regional Reference Centre for Microbiological Emergences at St.Orsola-Malpighi Hospital, Bologna, through the human surveillance, with the 2009 modality [7], to exclude WNV and USUV like possible cause. Moreover, the blood bags stored in the St. Orsola-Malpighi blood bank and collected in the presumed area of viral circulation during the period July 15-November 15, were tested by using nucleic acid amplification technique (NAAT) tests (PROCLEIX® WNV kit; Gen-Probe Inc., San Diego, CA, USA) [63].

The tests were conducted on human samples obtained from activity of the Regional Health Care System, according to the note of Regional Health Authority PG/2010/155768 of June 15 2010, so ethics approval was unnecessary. Human data were collected in the context of the standard diagnostic laboratory workflow in the framework of the Health, so that an implicit informed consent was obtained from each subject at the time of sample collection. These data were managed preserving the anonymity of patients.

### Virus Survey

RNAs present in samples were extracted using Trizol®LS Reagent (Invitrogen, Carlsbad, CA); cDNA synthesis was achieved using random hexamer (Roche Diagnostics, Mannheim, DE) and SuperScript® II Reverse transcriptase (Invitrogen, Carlsbad, CA) according to the manufacturers’ instructions.

Mosquito and bird samples were analyzed using three different polymerase chain reactions (PCRs): (1) traditional PCR, targeted to *NS5* gene fragment, for the detection of flavivirus-genus according to Scaramozzino *et al*. (2001) [64]; (2) traditional PCR for the detection of USUV [21]; and (3) real-time PCR for the detection of WNV, according to the method of Tang *et al*. (2006) [65]. Human samples were tested for WNV or USUV by real-time PCR assay based on specific TaqMan® probes [33], [65].

Fragments obtained by flavivirus-genus PCR were sequenced by an automated fluorescence-based technique following the manufacturer’s instructions (ABI-PRISM 3130 Genetic Analyzer, Applied Biosystems, Foster City, CA). In order to obtain WNV sequences, a traditional PCR protocol was performed on WNV-positive samples with the primers described in Lanciotti *et al.* (2000) [66], targeted to part of *C* and *prM* genes. For USUV, positive PCR products of both protocols were sequenced.

The obtained sequences were employed to perform a basic local alignment search tool (BLAST) in the GenBank library to confirm the specificity of positive reaction and to estimate the degree of identity of detected strains. The obtained sequences were compared with available GenBank sequences using the ClustalW alignment algorithm in the freeware program MEGA 5 [67] and a phylogenetic tree was produced by the same program.

### Statistical Analysis and Point Pattern Analysis

All the collected data were managed by a dedicated Microsoft Access database. To estimate the proportion of infected mosquitoes in *Cx. pipiens* samples, maximum likelihood estimation (MLE) was calculated using the Poolscreen2 program [68]. The data were analyzed by grouping together *Cx. pipiens* pools sampled in two weeks in the same province; only mosquitoes sampled at regularly sampled sites (plan traps) were considered. An MLE index was also evaluated for the weeks of collection of the WNV-positive pools sampled in Modena traps.

Land use characteristics of the sampling stations were obtained by overlaying georeferenced traps with the Emilia-Romagna Region Land Use 2008 layer in ArcGIS 9.2.

A 500 m radius buffer was constituted around each trap; the land uses were obtained for each of these areas and ranked according to five categories: urban area (residential area, commercial/industrial area), rural area (arable, farm), green area (gardens, urban parks, sport facilities), woodland (natural/artificial woods, orchards), wetland (swamps, marshes, quarries, river bank). Each station was assigned to the predominant land use category in the buffer area. The frequency of positive-PCR pools respect to the total number of *Cx. pipiens* pools sampled in different environments were compared via the χ^2^ test (p<0.05).

Normalized Difference Vegetation Index (NDVI) mean value of the sampling stations were obtained by overlaying 500 m radius buffer around each georeferenced traps with the MODIS/Terra images (MODIS/Terra Vegetation Indices 16-Day L3 Global 250 m SIN Grid V005– http://reverb.echo.nasa.gov/) in ArcGIS 9.2. Daily climatic data at 5×5 km grid resolution were provided by the Hydro-Meteorological Service of the Emilia-Romagna Regional Agency for Environmental Protection (ARPA-SIM) [69]. The total rainfall (Tr), potential evapotranspiration obtained by Hargreaves formula (Tev), hydroclimatic balance (IB = Tr-Tev), average temperature, minimum temperature (Tmin), maximum temperature (Tmax), temperature range (Tr = Tmax-Tmin), average relative humidity, minimum relative humidity and maximum relative humidity were acquired from June to September. The daily data from the plan trap squares were selected, and then the period data averages from June to September were calculated. Traps were classified as positive if at least one positive pool of USUV was obtained in 2009 or 2010 while traps were classified as negative if no positive pool was detected in the two years. The difference in the average of climatic data between positive and negative traps were compared using one-way ANOVA test with p<0.05. The average of the (abundance) of *Cx. pipiens* between positive and negative traps was also tested, as well as the same period average of obtained NDVI.

The abundance of mosquitoes collected between 2009 and 2010, was compared considering 14 plan traps (8 located in Bologna province and 6 in Ferrara province) and 6 Modena traps activated in the same period in 2009 and in 2010. Due to the not normal distribution of the dataset, the comparison was tested using non parametric test, Kruskal-Wallis test, with p<0.05.

A Nearest Neighbor Analysis (NNA) was conducted on total USUV-positive traps to identify spatial data patterns. Kernel density estimation (KDE) was applied at plan trap sites with USUV-positive mosquito pools. Bandwidth was 10 km, the same length as the grid in which plan traps were arranged. KDE was used as exploratory analysis to identify areas of intensive USUV circulation. NNA and KDE were performed using ESRI ArcGIS 9.2 and the Spatial Analyst extension.

The 95% volume contour of USUV KDE represents the boundary of the area that contains 95% of the volume of the obtained KDE distribution, and would therefore contain, on average, 95% of the points used to generate the KDE.

A quartile map was obtained of the hydroclimatic balance, the temperature range and minimum daily humidity recorded between June and September 2010 in the Emilia-Romagna region. Finally, percentages were calculated for the area within the 95% volume contour represented by the upper quartile of the temperature range and the by lower quartile of both the hydroclimatic balance and the minimum daily humidity. 

## Acknowledgments

We thank Katia Marzani and Jessica Milito (IZSLER) for their excellent laboratory assistance, Dr. Rodolfo Veronesi, Dr. Anna Medici, Dr. Gregorio Gentile, Dr. Roberto Pilani, Dr. Maurizio Magnani Dr. Arianna Puggioli (CAA) for mosquito collections.

## Supporting Information

Figure S1 **95% contour perimeter of USUV KDE (a); overlaid with quartile map of minimum relative humidity (b); from July to September 2010.**
(TIF)
