# Supplementary figures and images for: Mosquito, Bird and Human Surveillance of West Nile and Usutu Viruses in Emilia-Romagna Region (Italy) in 2010
Source: PLoS One. 2012 May 30;7(5):e38058. doi: 10.1371/journal.pone.0038058 (PMC3364206; doi:10.1371/journal.pone.0038058)

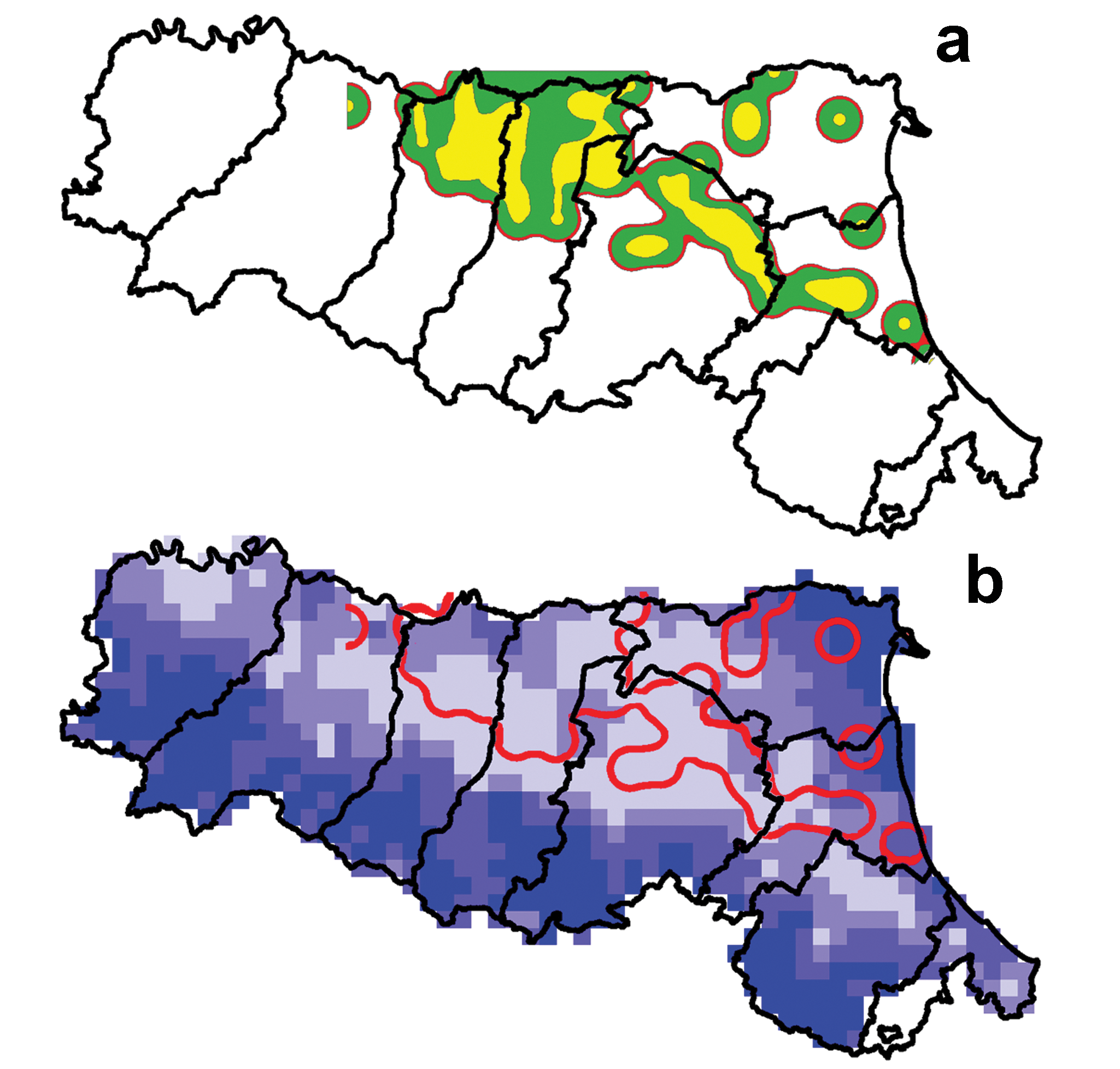

Supplement: Figure S1 — 95% contour perimeter of USUV KDE (a); overlaid with quartile map of minimum relative humidity (b); from July to September 2010. (TIF) [file pone.0038058.s001.tif]
